# Disseminated Nasal subtype Extranodal NK/T-cell lymphoma and its diagnostic difficulties in antemortem biopsies

**DOI:** 10.4322/acr.2023.445

**Published:** 2023-10-05

**Authors:** Aravind Sekar, Siddharth Jain, Jaimanti Bakshi, Suneel Rachagiri, Harish Bhujade, Rajender Kumar, Amanjit Bal

**Affiliations:** 1 Post Graduate Institute of Medical Education and Research, Department of Histopathology, Chandigarh, India; 2 All India Institute of Medical Sciences, Department of Medicine, New Delhi, India; 3 Post Graduate Institute of Medical Education and Research, Department of Otolaryngology, Chandigarh, India; 4 Post Graduate Institute of Medical Education and Research, Department of Radiodiagnosis, Chandigarh, India; 5 Post Graduate Institute of Medical Education and Research, Department of Nuclear Medicine, Chandigarh, India

**Keywords:** Hard Palate, Epstein-Barr Virus Infections, Autopsy

## Abstract

Extranodal NK/T- cell lymphoma (ENKTCL) is an aggressive lymphoma driven by Epstein-Barr virus (EBV) infection in genetically susceptible individuals. It was historically called a lethal midline granuloma. Due to the angio-destructive nature of ENKTCL, lymphoma cells are often accompanied and masked by necrosis and dense inflammation in the biopsy. Further, the biopsy may show vasculitis, which can mimic granulomatosis with polyangiitis. Due to these masquerades, ENKTCL is often misdiagnosed in the biopsy. Several biopsies may be required to establish the diagnosis. We describe the clinical course and autopsy findings of a young female who presented with a hard-palate ulcer. Antemortem biopsies failed to establish the diagnosis. The autopsy revealed an advanced nasal subtype of Extranodal NK/T-cell lymphoma with dissemination to the kidneys, adrenals, liver, spleen, and small intestine.

## INTRODUCTION

Extranodal NK/T-cell lymphoma (ENKTCL) is an extranodal lymphoma of NK or T- cell lineage driven by Epstein-Barr virus (EBV) infection in genetically susceptible individuals.^1^ It occurs predominantly in East Asia, Central and South America.^2-4^ Males are more commonly affected, and the median age of onset of the disease is 35-58 years.^5^ It is characterized by diffuse permeative growth of lymphoid cells with vascular damage, leading to extensive necrosis, ulceration of the overlying mucosa, and superimposed infections.

ENKTCL is divided into nasal and non-nasal types based on the primary site of the tumor.^6^ More than 80% of cases are of the nasal subtype, which primarily involves the nasal cavity, nasopharynx, oropharynx, and epiglottis initially and can erode the nasal floor, causing perforation of the hard palate. Due to its destructive nature, it was historically called a lethal midline granuloma.^7^ Other causes of midline destructive disease are granulomatosis with polyangiitis, sarcoidosis, IgG4-related disease, and several infectious causes such as syphilis, rhinoscleroma, mucormycosis, blastomycosis, tuberculosis, leprosy, and leishmaniasis.^8^

We describe the clinical course and autopsy findings of advanced nasal subtype Extranodal NK/T-Cell lymphoma, which masquerades as pseudoepitheliomatous hyperplasia, necrotizing inflammation, and vasculitis in antemortem biopsies.

## CASE REPORT

A 21-year-old female, apparently with no prior comorbidities, complained of fever over the last six months. Initially, the fever was high-grade and associated with chills and rigors but later became intermittent and of low-grade. One month after the fever's onset, she complained of left-sided nasal obstruction and stuffiness. She noticed a small, solitary painful ulcer on her hard palate, which gradually increased in size. Two weeks before admission, she developed rhinorrhea and nasal regurgitation of food and liquids; subtle swelling over her right cheek, nose, and around her right eye; and noticed a small perforation in her palate. There was, however, no history of bleeding, visible growth, or blackish discoloration. She lost 20 kg of weight in the last six months and lost appetite. There were no ear symptoms. There was no history of eye pain, decreased vision, redness, proptosis, meningism, seizures, altered sensorium, or focal neurological deficits. She initially visited the ENT outpatient department, where she was extensively worked up and given empirical antibiotics and fluconazole without improvement. She was admitted to the hospital due to symptomatic worsening. She had a history of left ear discharge three years ago and was treated. There was no similar illness in the family. There was no history of addiction to drugs, including cocaine. Bowel and bladder habits were regular. On general examination, she was conscious, oriented, anemic, and febrile (38,8^o^C). Her pulse rate was 130 beats per minute and regular, her blood pressure was 110/74 mmHg, and her SpO2 was 98%.

**Figure 1:**
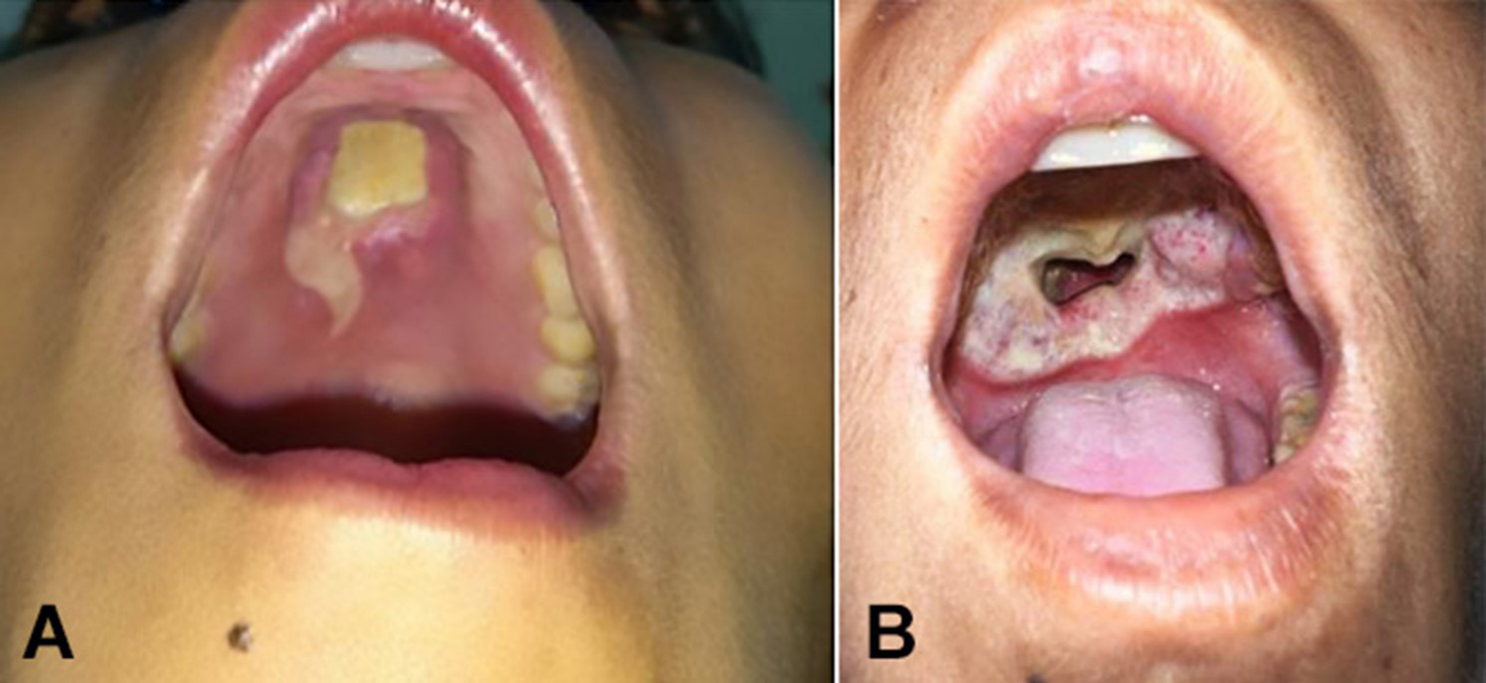
Oral cavity examination during initial hospital visits showing in **A -** an ulcer in the hard palate covered with yellowish slough, and in **B -** Deep ulcers with perforation and well-defined erythematous, edematous margins (B) were seen during later outpatient department (OPD) visits and during hospital admission.

Non-tender lymph nodes were palpable on the bilateral upper cervical region. The oral cavity showed a profusely foul-smelling ulcer on the hard palate, measuring 6 x 4 cm, with well-defined erythematous and edematous margins, yellowish sloughing on the surface, undermined edges, and an oro-nasal perforation (Figures 1A, and 1B).

Erythema, tenderness, and swelling over the nose, right cheek, and right periorbital area were present. The collapse of the nasal bridge with tenderness and nasal crusting was present on both sides of the nasal cavity. Nasal intonation was present. On otoscopic examination, one central and one marginal perforation without active discharge was seen in the left tympanic membrane. The bilateral external auditory canal was normal. Respiratory, cardiovascular, abdominal, and neurological examinations were normal.

**Figure 2:**
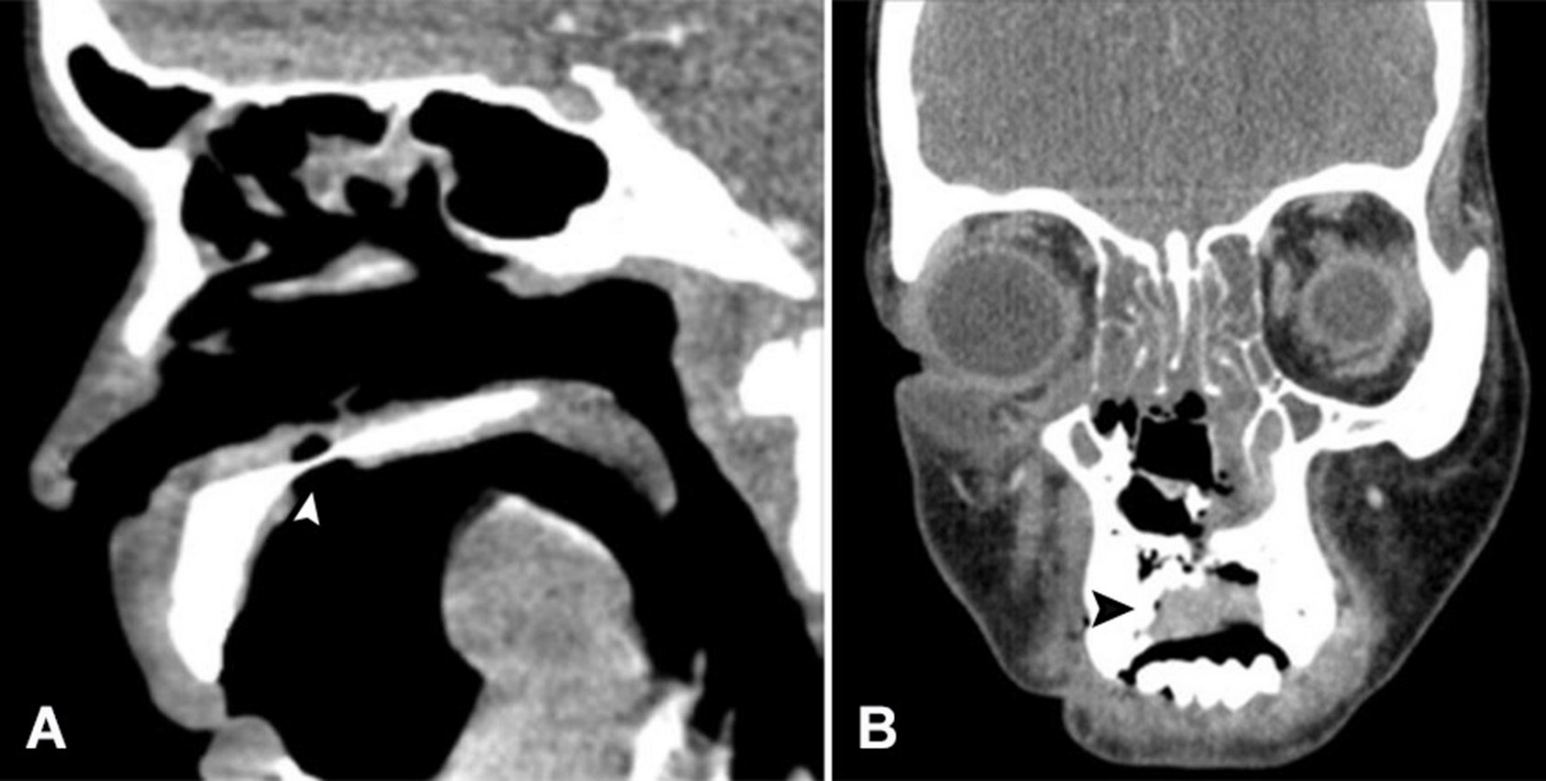
Contrast-enhanced Computed tomography showing in **A -** punched-out mucosal ulceration in the inferior and superior aspects of the hard palate (white arrowhead), and in **B -** Soft tissue thickening along the right lateral margin of the ulcer with rarefactions and erosions of adjacent bone (black arrowhead).

Contrast-enhanced Computed tomography (CECT) showed a punched-out mucosal ulceration in the inferior and superior aspects of the hard palate; soft tissue thickening in the bilateral maxillary, ethmoid, and frontal sinuses with soft tissue thickening along the right lateral margin with rarefactions and erosions of adjacent bone with suspicion of fungal or granulomatous diseases (Figures 2A, and 2B).

A PET-CT scan showed FDG uptake in the bilateral nasal sinuses, nasal cartilages, turbinates, and hard palate (with perforation and ulcer) with two lymph nodes in the cervical region. The largest lymph node measures 1.2 cm in maximum dimension and shows necrosis. The chest X-ray was normal during admission. A repeated thoracic examination, during the later course of the hospital stay, showed right lower zone haziness. An ultrasonogram of the abdomen shows mild cholecystitis and free fluid in the pelvis.

The laboratory findings at present admission showed hemoglobin of 7.2 gm/dl (reference range [RR]; 13.8-17.2 gm/dL); total leukocyte count of 12600 cells / mm^3^(RR; 4500-11000 cells/mm^3^); platelets of 250x10^3^ cells/mm^3^ (RR;150- 450x10^3^ cells/mm^3^); serum creatinine-1.1 mg/dL (RR;0.8-1.2 mg/dL); serum albumin-2.6mg/dL (RR; 3.4-5.4 mg/dL); serum total bilirubin-0.4 mg/dl(RR;0.3-1.0 mg/dl); CRP-224 mg/dl (RR;<1 mg/dl);Serum ferritin-1065 ng/ml (RR;12-150 ng/ml); pro calcitonin-0.34 ng/m; (RR;0.1 ng/ml); Fibrinogen-1.27 g/dl (RR;2-4 g/dl), D dimer-8165ng/ml (RR;< 250 ng/ml); PT/aPTT- 14.3/28.4 seconds (RR;10-12/30-40 seconds); Blood and urine culture was sterile. She was non-reactive to Human Immunodeficiency Virus. The *Treponema pallidum* hemagglutination test, the Mantoux test, and the serology for Hepatitis B and C were negative. Anti-neutrophil cytoplasmic antibodies for Proteinase-3 and Myeloperoxidase were negative two times. Six biopsies from the ulcer and the surrounding region were done during her OPD visits and admission. Three biopsies showed diffuse mucosa ulceration with dense mixed inflammatory cell infiltrates. Two biopsies showed hyperplastic changes in the squamous epithelium. One biopsy suggested the possibility of vasculitis. There were no atypical cells documented in any of these biopsies.

Based on the suggestion of vasculitis in one biopsy, a diagnosis of limited granulomatosis with polyangiitis was considered, and the patient planned for immunosuppression. However, with high spiking fever (40^o^C), leukocytosis, and extensive foul-smelling yellowish slough, the possibility of secondary infection was kept. She was empirically started on intravenous piperacillin, tazobactam, and clindamycin, later upgraded to meropenem and teicoplanin after 72 hours because of persistent fever. ENT and radiotherapy consultations were taken, and a high possibility of lethal midline granuloma was considered, with a possible need for radiotherapy. A submental lymph node biopsy was done, which showed necrotizing lymphadenitis. Endoscopic debridement of necrotic palate-nasal tissue with bilateral middle meatal antrostomy was done. Steroids were added (hydrocortisone 50 mg q8h) under cover of ongoing antibiotics. The patient became afebrile after debridement. Debrided tissue showed Gram-negative bacteria, but cultures were sterile. She remained afebrile for a few days, and the institution of definitive therapy was planned. However, she developed a recurrence of high-grade fever. Antibiotics were upgraded (colistin and vancomycin), but the patient developed worsening hypotension, requiring increasing inotropic support. She developed sudden onset breathing difficulty and profuse sweating. On evaluation, she had a blood pressure of 70/50 mm Hg, a poor GCS (E3V1M3), and hypoglycemia (20 mg/dL). Despite hypoglycemia correction, she continued deteriorating and had a refractory shock with severe metabolic acidosis on arterial blood gas analysis. Finally, she sustained a cardiac arrest from which she could not be revived.

## AUTOPSY FINDINGS

A partial autopsy was performed with a midline thoracoabdominal incision with the consent of the deceased relatives. Hard palate and paranasal sinus tissue were not sampled during autopsy as dissector could not be able to open the mouth due to rigor mortis. Serous cavities were within normal limits.

Both lungs weighed 649 g (RR, 550-650 g). The pleural surface was shiny, and both lungs' cut surfaces were sub-crepitant to feel without focal lesions. A few hilar lymph nodes were enlarged. Microscopy showed diffuse pulmonary edema.

**Figure 3:**
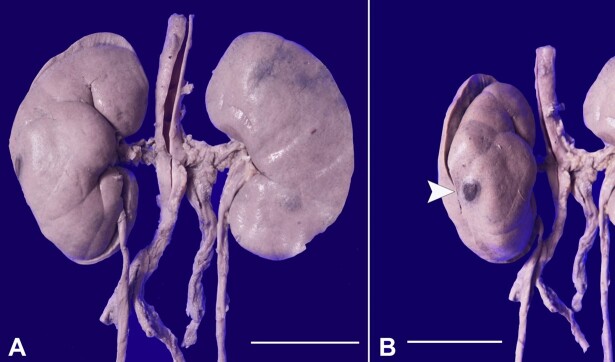
Gross photograph of bilateral kidneys showing in **A -** blotchiness and blackish nodular lesions on the capsular surface (scale bar 4 cm), **B -** Nodules are hemorrhagic with central necrosis on closer examination (scale bar 6 cm) (white arrowhead).

Both kidneys weighed 360 g (RR, 160-320 g). The capsular surface of both kidneys showed blotchiness and a few blackish nodular lesions measuring 1 to 1.5 cm in maximum dimension (Figures 3A, and 3B).

**Figure 4:**
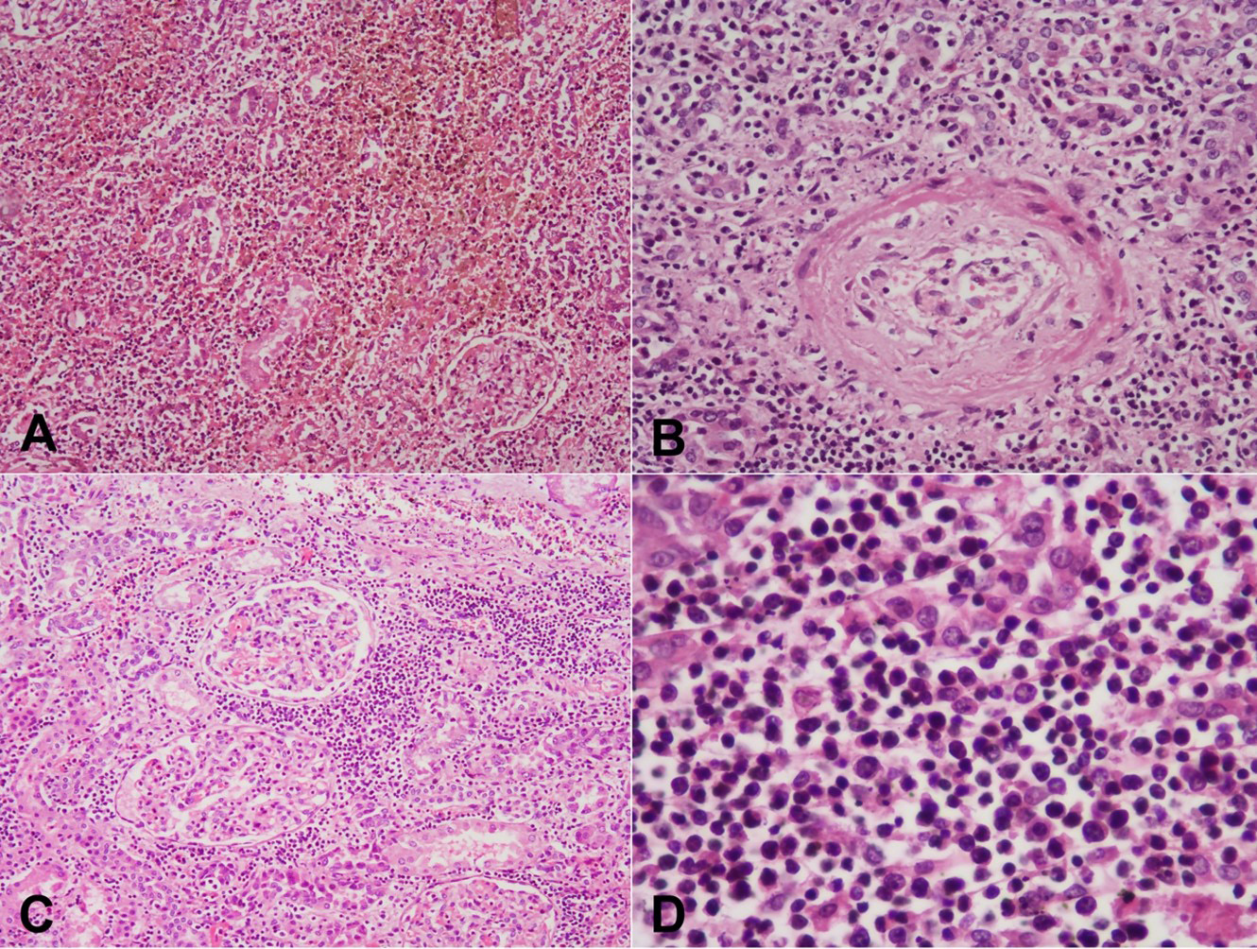
Photomicrography of the kidney lesion showing in **A -** hemorrhage, debris, and lymphoid cells in the tubulointerstitial compartment, **B -** with evidence of vasculitis in one of the interlobular arteries; **C -** Lymphoid cells are insinuating in between the tubules and **D -** have an irregular nuclear membrane, folded hyperchromatic nuclei, and a moderate amount of pale cytoplasm.

Nodules were soft in consistency, with central focal necrosis and hemorrhagic on the cut surface. Microscopy of these nodular lesions showed hemorrhage, necrotic debris, and dense infiltrates destroying the renal parenchyma (Figure 4A). The lesion’s entrapped interlobular arteries entrapped showed evidence of vasculitis with fibrinoid necrosis and debris in their walls (Figure 4B). On higher magnification, the infiltrates were composed of atypical lymphoid cells insinuating between the tubules and causing destruction (Figure 4C). Atypical lymphoid cells have an irregular nuclear membrane, folded hyperchromatic nuclei, and a moderate amount of pale cytoplasm (Figure 4D).

**Figure 5:**
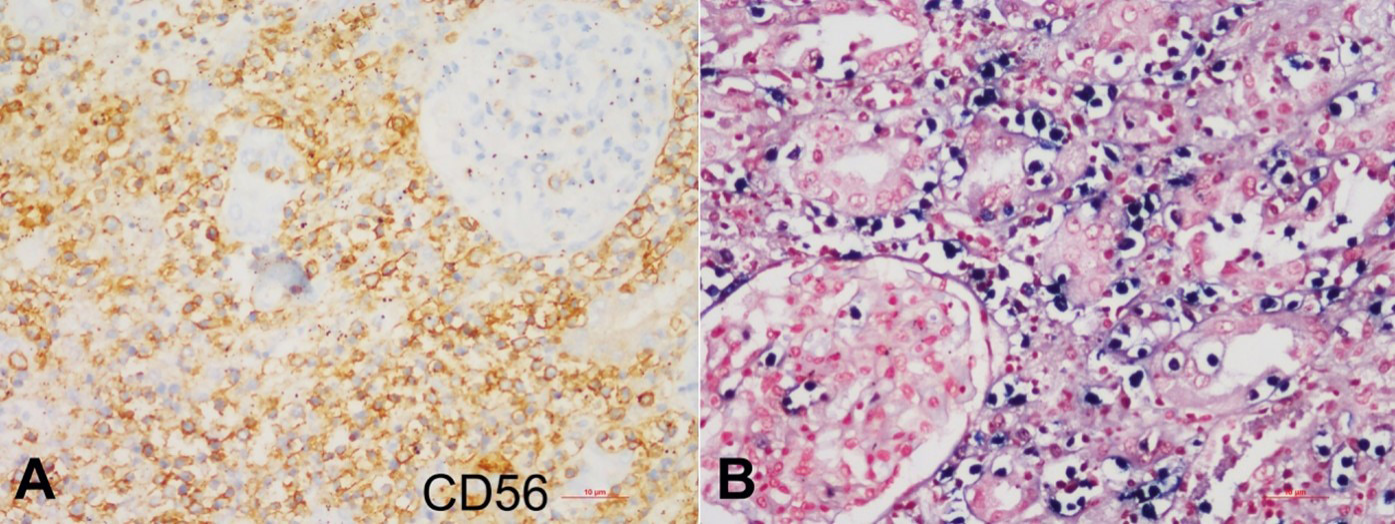
Photomicrographs of the kidney lesion. **A -** immunohistochemistry (IHC), lymphoid infiltrates show intense membranous positivity for CD 56; **B -** In-situ hybridization for EBV-encoded RNA (EBER) exhibits the presence of Epstein-Barr virus in most of the lymphoma cells.

On immunohistochemistry (IHC), lymphoid infiltrates were negative for CD20; positive for CD3 and CD3 epsilon in scattered cells in the background; and intense membranous positivity for CD56, confirming NK/T- cell origin (Figure 5A).

Further, in-situ hybridization for EBV-encoded RNA (EBER) demonstrates the Epstein-Barr virus’s presence in most lymphoma cells. (Figure 5B). Glomeruli did not show any proliferative lesions or segmental sclerosis. Arteries in the other areas were normal.

**Figure 6:**
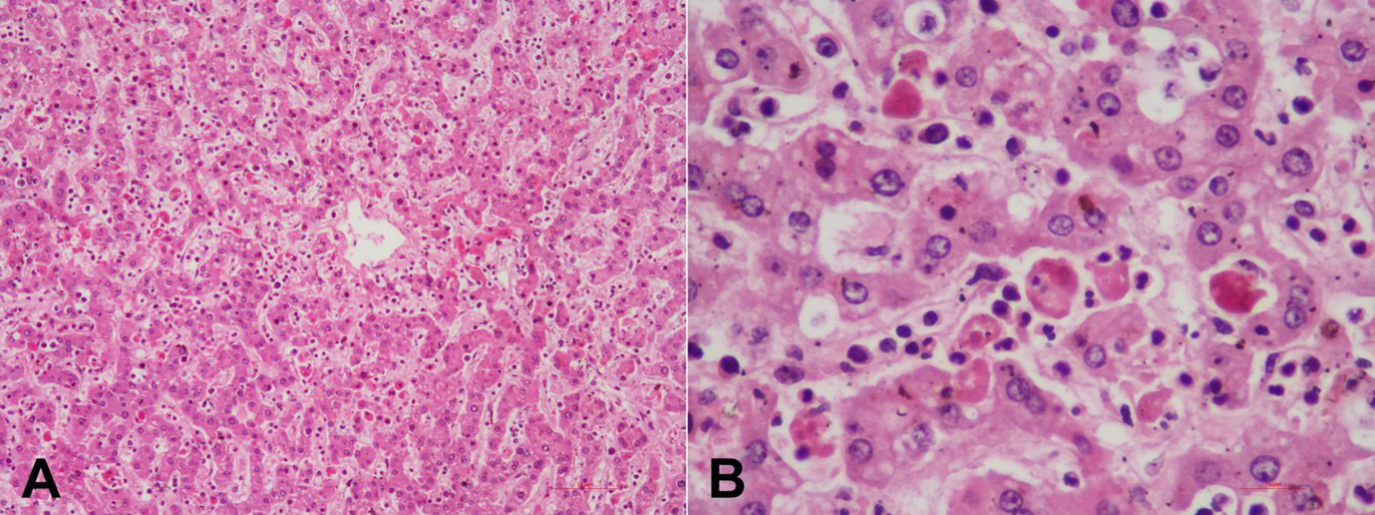
Photomicrographs of the liver show in **A -** sinusoidal infiltration by lymphoma cells; **B -** significant increase in the number of histiocytes showing hemophagocytosis (B). (H&E; **A -** 100x; **B -** 400x).

The liver weighed 1100 g (RR; 775-2395 g). The capsular and cut surfaces were unremarkable, without focal lesions. Microscopy showed maintained lobular architecture. The sinusoids were busy and showed infiltration by NK/T- cell lymphoma (Figure 6A) and striking hemophagocytosis (Figure 6B). The spleen weighed 512 g (RR; 51-275 g). The cut surface shows congestion. Microscopy shows infiltration by NK/T-Cell lymphoma and significant hemophagocytosis. The small intestine and adrenals also showed infiltration by NK-cell lymphoma cells.

Lymph nodes showed maintained nodular architecture with a prominence of sinus histiocytosis with occasional hemophagocytosis. No atypical cells were seen. The uterus, ovaries, bladder, and pancreas were normal.

**Figure 7:**
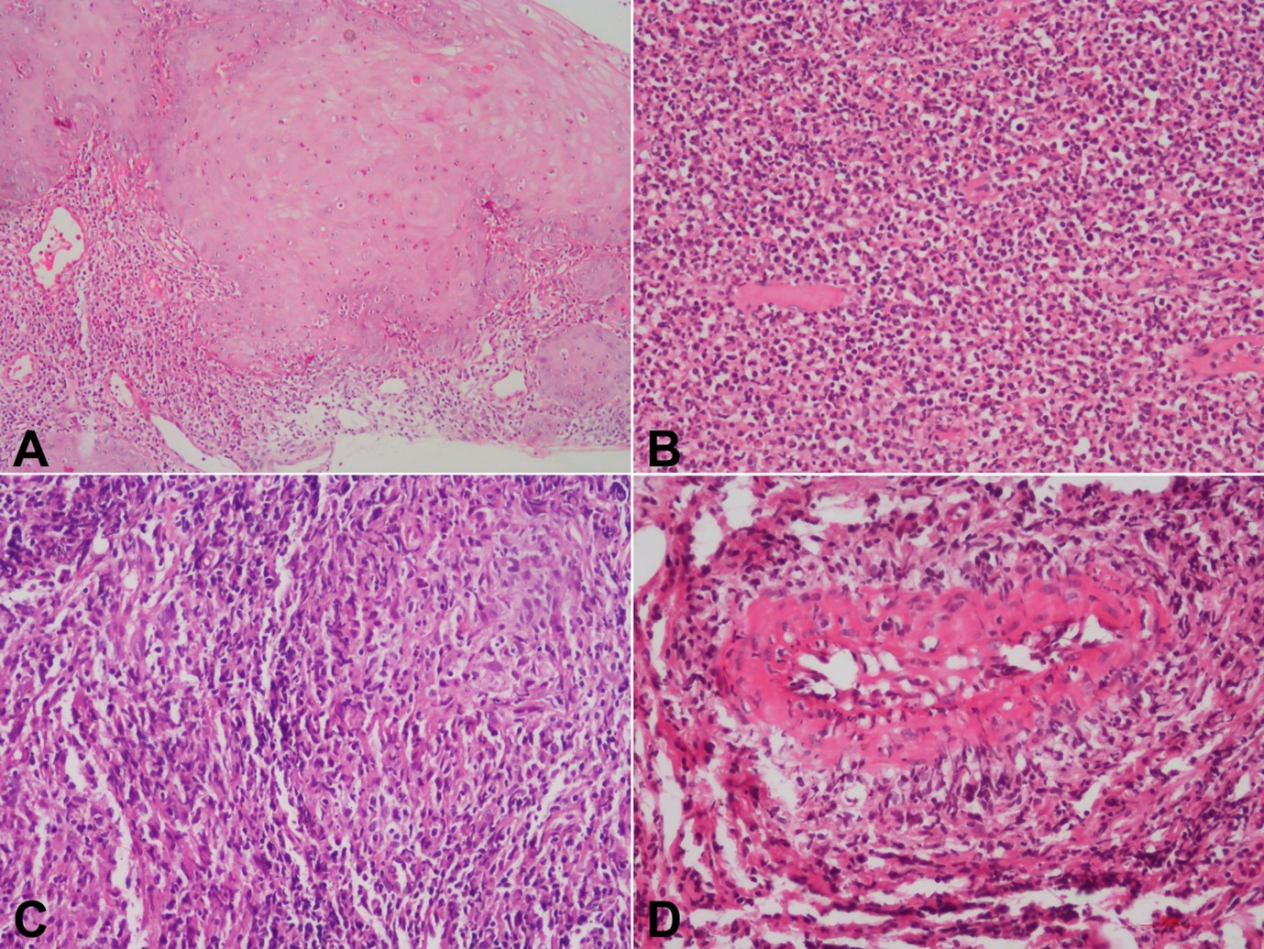
Photomicrographs antemortem biopsies showing in **A -** pseudo-epitheliomatous hyperplasia; **B -** granulation tissue with dense inflammation; **C -** vague granuloma formation; **D -** arteritis (H&E, A to C 200X, D 400X).

All six antemortem biopsies were reviewed. They showed pseudoepitheliomatous hyperplasia (Figure 7A) in two biopsies, granulation tissue with necrotic debris, and dense neutrophil-rich infiltrates (Figure 7B) in three biopsies; vague epithelioid cell granulomas (Figure 7C) in one biopsy; vasculitis with debris in the arterial wall in one biopsy (Figure 7D). However, in one biopsy CD 56 immunostaining highlighted the occasional lymphoma cells in a dense, mixed inflammatory cell background.

## DISCUSSION

The ENKTCL-nasal subtype is the prototype of lymphomas driven by EBV.^9^ EBV, a gamma herpesvirus with a tropism for B cells. It infects more than 90% of the population worldwide and remains latent in memory B cells. HLA-DPB1, HLA-DRB1, and IL18 RAP are the common predisposing haplotypes observed in East Asia.^10^, Various external and immune factors, in genetically susceptible individuals, trigger virus activation and transmission into T-cells and NK-cells. Another mechanism through which transmission can occur is when NK or T-cells attempt to kill EBV-infected B cells. The transmission further stimulates the development of various reactive and neoplastic lymphoproliferative disorders. It includes severe mosquito bite allergy, hydroa vacciniforme lymphoproliferative disorder, systemic chronic active EBV disease, systemic EBV-positive T-cell lymphoma of childhood, and nodal and extranodal NK/T-cell lymphoma.^11^

Though the exact pathogenesis is unknown, the constitutive activation of the JAK/STAT pathway through mutation plays an essential role in the development of ENKTCL.^12^ Due to their angio-destructive nature, necrosis and intense inflammation frequently accompany and conceal lymphoma cells.^13^ It may lead to misdiagnosis or non-diagnosis, as in the index case, which could not be made even after six biopsies. In Y-T Lee et al.^14^ a case study, ENKTCL was diagnosed at the eighth biopsy.

Clinically, the important differential diagnosis is granulomatosis with polyangiitis (GPA) with sino-nasal involvement.^14^ Septal perforation and saddle nose deformity can occur in 10-30% of GPA cases.^15^ Although 80% of individuals with GPA will have renal involvement within two years, only 10-20% of patients with GPA have renal manifestation at the time of diagnosis.^16^ Further, anti-neutrophil cytoplasmic antibodies can be negative in 30-40% of limited GPA cases.^17^ Differentiating GPA from the ENKTCL-nasal subtype is challenging even on superficial biopsies, as both diseases cause vasculitis and extensive necrosis. Vasculitis in ENKTCL may be caused by the angiocentric and/or angioinfiltrative nature of lymphoma cells or cytotoxic substances produced by lymphoma cells. A thorough biopsy analysis to recognize the presence of atypical cells in and around the arterial wall helps in the diagnosis of ENKTCL. Further, immunomarker for Natural killer cells such as CD56 and in situ hybridization for Epstein- Barr virus encoded RNAs can be done.

In the advanced stage, systemic spread can occur. In the index case, ENKTCL disseminated to the bilateral kidneys, adrenals, liver, spleen, and small intestine. In addition, marked hemophagolymphohistiocytosis (HLH) was noted in the liver, spleen, and lymph nodes. HLH can be secondary to EBV infection or due to the systemic spread of ENKTCL. EBV is the most common cause of secondary HLH.^18^ After it and other viruses; lymphomas account for 30% of secondary HLH. High serum concentrations of IL-18, TNF-alpha, and IFN-gamma in ENKTCL patients can activate macrophages, cause hemophagocytosis, and result in poor survival outcomes.^19^

## CONCLUSION

EBV-positive Extranodal NK/T- cell lymphoma is a rare entity with high aggressiveness and a poor prognosis. Due to its angio-destructive properties and secondary infection, it may mimic inflammatory lesions or vasculitis in the biopsy. Multiple biopsies are often required to establish the diagnosis. Clinical vigilance, in-depth analysis of the biopsy for atypical lymphoid cells, performing CD56 immunostaining, and In-situ hybridization for Epstein-Barr virus-encoded RNA will aid in diagnosing ENKTCL.

## References

[1] Li W, Li W (2022). Leukemia.

[2] Yoon SO, Suh C, Lee DH (2010). Distribution of lymphoid neoplasms in the Republic of Korea: analysis of 5318 cases according to the World Health Organization classification. Am J Hematol.

[3] Yang QP, Zhang WY, Yu JB (2011). Subtype distribution of lymphomas in Southwest China: analysis of 6,382 cases using WHO classification in a single institution. Diagn Pathol.

[4] Quintanilla-Martinez L, Franklin JL, Guerrero I (1999). Histological and immunophenotypic profile of nasal NK/T cell lymphomas from Peru: high prevalence of p53 overexpression. Hum Pathol.

[5] Au WY, Weisenburger DD, Intragumtornchai T (2009). Clinical differences between nasal and extranasal natural killer/T-cell lymphoma: a study of 136 cases from the International Peripheral T-Cell Lymphoma Project. Blood.

[6] Kim SJ, Yoon DH, Jaccard A (2016). A prognostic index for natural killer cell lymphoma after non-anthracycline-based treatment: a multicentre, retrospective analysis. Lancet Oncol.

[7] Mendenhall WM, Olivier KR, Lynch JW, Mendenhall NP (2006). Lethal midline granuloma-nasal natural killer/T-cell lymphoma. Am J Clin Oncol.

[8] Borges A, Fink J, Villablanca P, Eversole R, Lufkin R (2000). Midline Destructive Lesions of the Sinonasal Tract: Simplified Terminology Based onHistopathologic Criteria. AJNR Am J Neuroradiol.

[9] Montes-Mojarro IA, Fend F, Quintanilla-Martinez L (2021). EBV and the Pathogenesis of NK/T Cell Lymphoma. Cancers (Basel).

[10] Li Z, Xia Y, Feng LN (2016). Genetic risk of extranodal natural killer T-cell lymphoma: a genome-wide association study. Lancet Oncol.

[11] Kim WY, Montes-Mojarro IA, Fend F, Quintanilla-Martinez L (2019). Epstein-Barr Virus-Associated T and NK-Cell Lymphoproliferative Diseases. Front Pediatr.

[12] de Mel S, Hue SSS, Jeyasekharan AD, Chng WJ, Ng SB (2019). Molecular pathogenic pathways in extranodal NK/T cell lymphoma. J Hematol Oncol.

[13] Xiang CX, Chen ZH, Zhao S (2019). Laryngeal extranodal nasal-type natural killer/t-cell lymphoma: a clinicopathologic study of 31 cases in China. Am J Surg Pathol.

[14] Lee YT, Chang YS, Lai CC, Chen WS, Yang AH, Tsai CY (2012). Natural killer (NK)/T-cell lymphoma mimicking granulomatosis with polyangiitis (Wegener’s). Scand J Rheumatol.

[15] Coordes A, Loose SM, Hofmann VM (2018). Saddle nose deformity and septal perforation in granulomatosis with polyangiitis. Clin Otolaryngol.

[16] Sinico RA, Di Toma L, Radice A (2013). Renal involvement in anti-neutrophil cytoplasmic autoantibody associated vasculitis. Autoimmun Rev.

[17] Sharma A, Naidu GSRSNK, Rathi M (2018). Clinical features and long-term outcomes of 105 granulomatosis with polyangiitis patients: A single center experience from north India. Int J Rheum Dis.

[18] Marsh RA (2018). Epstein-Barr virus and hemophagocytic lymphohistiocytosis. Front Immunol.

[19] Lim SW, Ryu KJ, Lee H, Ko YH, Kim WS, Kim SJ (2019). Serum IL18 is associated with hemophagocytosis and poor survival in extranodal natural killer/T-cell lymphoma. Leuk Lymphoma.

